# Assessment of exposure to piroplasms in sheep grazing in communal mountain pastures by using a multiplex DNA bead-based suspension array

**DOI:** 10.1186/1756-3305-6-277

**Published:** 2013-09-24

**Authors:** Amaia Ros-García, Jesús F Barandika, Ana L García-Pérez, Ramón A Juste, Ana Hurtado

**Affiliations:** 1 Department of Animal Health, NEIKER - Instituto Vasco de Investigación y Desarrollo Agrario, Berreaga 1, Derio, Bizkaia 48160, Spain

**Keywords:** *Babesia*, *Theileria*, Luminex, Suspension microarray, Sheep, RLB, Tick-borne pathogens, Piroplasmosis

## Abstract

**Background:**

Piroplasms are tick-borne hemoprotozoans with a major impact on extensive management systems. Detection of sub-clinical low-level carriers, which can act as source of infection for vector ticks, is key to protect livestock trade and facilitate preventive control programs. The purpose of this study was to develop a method for the detection of ovine piroplasms and to use it in a field study aimed at investigating piroplasms infection in semi-extensive production systems in the Basque Country (northern Spain).

**Methods:**

A DNA bead-based suspension array using the Luminex® xMAP technology that included a generic *Theileria*-*Babesia* control probe, 6 species-specific probes, and an internal control probe was developed to detect and identify piroplasms that infect sheep. To monitor piroplasm infection in clinically healthy sheep from 4 flocks that share communal mountain pastures, blood samples were collected during 2 grazing seasons.

**Results:**

Piroplasms were detected in 48% (214/446) of blood samples, nearly half of them (49.1%, 105/214) as mixed infections. Five different piroplasms were identified: *Theileria* sp. OT3 in 34.8% of the samples, *Theileria ovis* in 20.9%, and at lower prevalences *Babesia motasi* (12.3%), *Theileria luwenshuni*/OT1 (10.5%) and *Babesia ovis* (6.3%). Despite differences among flocks associated to differences in management, an increasing trend in the incidence of piroplasm infection with increasing age of animals after increased tick exposure was observed. This increment could be attributed to continued re-infection associated with re-exposure to ticks at grazing. Ticks were collected from animals (4 species) and vegetation (8 species), and associations between tick abundance seasonality and risk of infection with the different piroplasms were established.

**Conclusion:**

The multiplex Luminex® xMAP procedure is a rapid and high throughput technique that provided highly specific and sensitive identification of single and mixed piroplasm infections in blood of sheep carriers. This study confirmed a situation of endemic stability for piroplasm infection in the region, where infection is present in the absence of clinical signs, and mountain grazing allows for sufficient inoculation rates to maintain such situation.

## Background

*Theileria* and *Babesia* species are tick-borne haemoprotozoan parasites that infect livestock and wildlife in tropical and sub-tropical regions of the world, including sheep. The impact of piroplasmosis is higher on management systems where animals spend long periods grazing in mountain pastures exposed to tick bites. In the Basque Country (northern Spain) there are approximately 324,000 sheep, of which 90% are Latxa breed, the native dairy sheep of the Basque Country, whose production characteristics have been reported elsewhere [1]. Animal husbandry is semi-extensive; sheep are kept on farmland pastures from winter to early spring for lambing (one lambing per ewe per year) and milking, and on communal mountain pastures otherwise. Milking starts after the lamb(s) are slaughtered or weaned, and milked ewes are normally dried off at the beginning of the summer with total lactation length (from lambing to drying off) of about five months. Most of milk is processed to Idiazabal cheese. A previous study carried out in the region [2] showed a relatively high prevalence of sub-clinical infections in the sheep population and identified five different piroplasms: *Babesia ovis*, *Babesia motasi*, *Theileria ovis*, *Theileria* sp. OT1 and *Theileria* sp. OT3. While *B. ovis* and *B. motasi* are considered highly and moderately virulent species, respectively, the *Theileria* species found are considered to be less virulent or benign [3,4]. *Theileria* sp. OT1 shares 99.6% similarity in the 18S rRNA gene with a *Theileria* sp. recently described in China (*Theileria luwenshuni*) considered highly virulent [5]. *Theileria* sp. OT1 is widespread in the Basque Country among healthy sheep and is considered non-pathogenic [2].

Animals that survive acute infection generally become low-level carriers of the parasites, and could remain persistently infected for years without apparent clinical signs [3,4]. Ticks feeding on these sub-clinical carriers can become infected. Ticks of the genera *Rhipicephalus* and *Haemaphysalis* are considered the vectors for *T. ovis*, *B. ovis* and *B. motasi*[3,4]. For *T. luwenshuni* the principal vector in China seems to be *Haemaphysalis qinghaiensis*[6], whereas the vector of *Theileria* sp. OT3 is as yet unknown. *Theileria lestoquardi*, a highly virulent *Theileria* not reported in Spain, is transmitted by *Hyalomma* spp. [3], a tick genus very rarely found in the Basque Country. In *Babesia* spp., there is both transovarial and transstadial transmission, whereas in *Theileria* spp. only transstadial transmission occurs [3,7]. Also, whereas most *Babesia* spp. inoculated by ticks invade erythrocytes of the vertebrate host directly, *Theileria* species first infect lymphocytes leading to clonal expansion of the infected cells before invading erythrocytes [8].

Carrier animals are known to exhibit fluctuating low parasitaemia which sometimes even escapes detection. This is particularly true for *Babesia* spp. infection where intraerythrocytic piroplasms in the circulating bloodstream rarely exceed a few percent even during acute infection [9,10]. Thus, detection of sub-clinical carriers requires sensitive diagnostic tools able to detect and identify the different piroplasms that infect sheep. Reverse Line Blot (RLB) has been widely used for this purpose [2,11,12]. However, despite its multiplexing capacity, RLB is long and tedious. Here, we developed a DNA bead-based suspension array test based on the Luminex® xMAP technology to detect and differentiate ovine *Babesia* and *Theileria* species. This technique has already been shown to provide higher sensitivity and throughput than RLB [13]. Once developed, we used it to monitor piroplasms infection among clinically healthy sheep grazing in a communal mountain pasture in a region where sub-clinical piroplasm infection was detected in the past [2]. Ticks from animals and vegetation were also collected to study piroplasms in relation with tick abundance and distribution.

## Methods

### Animal selection and sample collection

The study was carried out in a communal mountain pasture (700–1,200 m above sea level) located in Alava, a province located in the Basque Country, northern Spain. Climate is of transitional type, resulting from the interaction of an Atlantic climate and a Continental Mediterranean climate, with cold winters and mild summers. Mean annual rainfall is *ca.* 900 mm. Vegetation is mainly composed of beech trees (*Fagus sylvatica*) with areas of pines (*Pinus* spp.) and small patches of oaks (*Quercus* spp.) interspersed with patches of natural grass.

Four flocks of Latxa breed (F1, F2, F3 and F4) that graze in these communal mountain pastures were monitored for piroplasms infections: flocks F1 & F2 during two grazing seasons (2011–2012), flock F3 solely in 2011, and flock F4 in 2012. There were some differences among flocks regarding milk production and management. Thus, in F1 and F2 management was more intensive and milk production was clearly higher than in F3 and F4. The use of communal mountain pastures was also different among flocks. In this way, in F1 and F2 only a few animals (non-milking ewes) grazed in these pastures from April to middle of July, whereas in F3 and F4 a higher number of animals (non-milking ewes, replacement lambs and their dams) were kept on mountains in this period of the year. From middle of July until November all animals of the four flocks grazed in mountain pastures. While grazing in the mountains, no feed supplementation was given and feeding relied only on pasture availability. No specific treatments against ticks were carried out in any of the flocks. Size of flocks ranged from 120 to 599 animals.

All flocks were sampled three times per year, before going to the communal pastures (spring), and twice during the grazing season (summer and autumn). Blood samples were randomly collected from *ca.* 25 animals per flock and sampling, divided into three age categories (A, <1 year; B, 1–2 years; C, >2 years). Animals were apparently healthy at sampling. Thus, a total of 446 blood samples were collected from clinically healthy sheep aged 2 months - 8 years. During the second year sampled animals were inspected for the presence of ticks, mainly on the ears, along the nape of the neck, perineum, udder and tail base, which were manually removed, counted and identified [14,15].

### Questing ticks sampling

To investigate tick abundance in the same mountain pastures where animals graze, ticks were monthly collected between May 2011 and April 2013 from the vegetation by blanket dragging a 1 m^2^ white cotton towel over vegetation transects of 100 m, stopping every 10 m to collect and count all attached adult and nymph ticks. For larvae counts, towels were examined at the laboratory and counted; exceptionally, when the amount of collected larvae was too large, an estimate was made after visually dividing the towel into 10 cm stripes and counting three of them (first, middle and last). Ticks were identified using taxonomic keys [14,15] and stored at −20°C. To compare the abundance of each tick species and stages between samplings, tick count data were converted into tick abundance indexes (TAI) for larvae, nymphs and adults, which express the number of each tick species and stage collected in transects of 100 m^2^, as TAI = TR x 100 / *a*, where TR is the number of ticks recorded and *a* is the sampled area in square meters.

### DNA extraction

DNA was extracted from 200 μl of ovine blood using the QIAamp DNA Mini Kit (Qiagen, Hilden, Germany), including negative extraction controls every 10 samples. DNA yields were determined with a Nanodrop® ND-1000 Spectrophotometer (Nanodrop Technologies, DE, USA), and DNA was stored at *−*20°C until subsequent analysis.

### DNA amplification

PCR was used to amplify the hypervariable V4 region of the 18S rRNA gene of the genera *Babesia* and *Theileria* using primers RLB-F2 (phosphorylated-5’-GACACAGGGAGGTAGTGACAAG-3’) and RLB-R2 (biotinylated-5’-CTAAGAATTTCACCTCTGACAGT-3’) (Sigma–Aldrich, MO, USA) as reported before [16]. An internal amplification control (IAC), which is co-amplified with the same primers as the piroplasms DNA, was also included in each tube [13].

PCR amplification was performed in a final volume of 50 μl containing 50 ng of genomic DNA, 200 nM of each primer, 1 × PCR buffer, 1.5 mM MgCl_2_, 200 μM of each dNTP, 10 copies of the internal amplification control (IAC) plasmid and 1U of Taq Platinum Polymerase (Invitrogen, CA,USA). PCR conditions consisted of an enzyme activation step of 4 min at 94°C, and 40 cycles of 30 s at 94°C, 1 min at 51°C and 35 s at 72°C. Extraction controls and PCR negative (water) controls were included in each PCR reaction as negative controls.

### Molecular detection and identification of ovine piroplasms by Luminex® xMAP technology

#### Luminex procedure description

Eight capture probes were included in the microsphere-based Luminex detection procedure (Table 1). Four of them were as previously designed for RLB hybridisation (the *Theileria*-*Babesia* conserved catch-all TB probe, *T. ovis*, *Theileria* sp. OT1, *Theileria* sp. OT3). Since *Theileria* sp. OT1 shares 99.6% similarity in the 18S rRNA gene with *Theileria* sp. China 1, recently named *T. luwenshuni*, the probe for *Theileria* sp. OT1 would also hybridise with *T. luwenshuni* and will therefore be referred as *T. luwenshuni*/OT1 hereafter. Three probes (*B. ovis*, *B. motasi* and *T. lestoquardi*) were newly designed using PrimerPlex software (PREMIER Biosoft International, CA, USA) for the Luminex assay. A further probe designed to detect the IAC in the Luminex assay was also included [13].

**Table 1:** Oligonucleotide probes used in the Luminex suspension array for the detection and species identification of ovine piroplasms

**Probe**	**Sequence (5’- 3’)**	**Size (bp)**	**nmol**	**References**
Catch-all TB	TAATGGTTAATAGGA(A/G)C(A/G)GTTG	22	1	[11]
*Babesia ovis*	CGCGGCCTTTGCGTTACTTT	20	0.5	This work
*Babesia motasi*	TCCGTTATTGGAGTATTGCG	20	0.5	This work
*Theileria ovis*	TTTTGCTCCTTTACGAGTCTTTGC	24	0.5	[1]
*Theileria luwenshuni*/OT1	ATCTTCTTTTTGATGAGTTGGTGT	24	0.5	[1]
*Theileria* sp. OT3	ATTTTCTCTTTTTATATGAGTTTT	23	0.5	[1]
*Theileria annulata / lestoquardi*	GGGTCTGTGCATGTGGCTTTT	20	0.5	[8]
IAC	GCATCGGTTACAAGAACGCA	20	0.5	[8]

Catch-all TB, *Theileria*-*Babesia* conserved probe; *IAC*, internal amplification control.

The capture probes were bound to different polystyrene microsphere sets as described elsewhere [13]. Biotinylated PCR products were hybridised to microspheres coupled with the probes mentioned above in 96-well plates in a suspension array. The optimal hybridisation temperature and incubation time proved to be 54°C and 30 min with shaking at 650 rpm in a Thermomix Comfort (Eppendorf). Detection was carried out by incubation at 54°C during 10 min with agitation at 650 rpm using streptavidin-phycoeritryn in 1x TMAC buffer. Finally, the signals produced for each bead were analyzed using the Luminex 200® system (Austin, TX) and expressed as mean fluorescent intensity (MFI) values. The cut-off value for a positive result was calculated for each assay and probe as described elsewhere [13].

#### Specificity, sensitivity and test performance

The analytical specificity of the probes was tested against 27 recombinant plasmids constructed as previously described [13] and containing inserts corresponding to the V4 variable region of the 18S rRNA gene of the following *Theileria* spp. and *Babesia* spp. from sheep, cattle, horse, dogs and wildlife: *T. ovis, T. lestoquardi, Theileria* sp. OT1, *Theileria* sp. OT3, *B. ovis, B. motasi, Theileria annulata, Theileria buffeli, Theileria parva, Babesia bovis, Babesia divergens, Babesia bigemina, Babesia major, Babesia occultans, Theileria equi* (genogroups A-D)*, Babesia caballi* (genogroups A and B)*, Babesia canis, Babesia vogeli, Babesia gibsoni, Theileria annae, Babesia microti, Babesia* sp. EU1, *Theileria* sp*.* 3185/02*.*

Analytical sensitivity of the hybridisation assay was assessed by processing serially diluted plasmids (10^3^–1 copies) of the ovine *Theileria* spp. and *Babesia* spp. To assess the diagnostic test performance of the Luminex assay, 177 clinical samples from sheep were analysed in parallel by RLB hybridisation, which was performed as described elsewhere [2]. To determine the detection limit of the Luminex assay, blood from an animal with clinical symptoms of piroplasmosis was collected. Haematological examination revealed low values in red blood cells (8.42 x 10^6^ erythrocytes/mm^3^), low haematocrit (22%) and leukopenia (2.7 x 10^3^ leukocytes/mm^3^). Giemsa-stained slides were examined under oil immersion using × 100 objective lens and 1.16% of the erythrocytes were parasitized by *B. ovis*. The number of erythrocytes and intracellular forms with morphology compatible with *B. ovis* present in 10 microscopic fields were counted. Mean parasitaemia was set at 0.0168 babesias/erythrocyte and parasitaemia estimated at 1.4 x 10^5^ babesias/mm^3^. Blood was serially diluted (1/10 dilutions until 1.4 babesias/mm^3^, and 1/2 dilutions thereafter) and processed as described above. The V4 hypervariable region of the last positive dilution was sequenced using the ABI BigDye™ Terminator Cycle Sequencing Ready Reaction Kit and an ABI3130 Genetic Analyzer (Applied Biosystems, Foster City, CA, USA).

### Statistical analysis

The level of agreement between methods (RLB and Luminex performed in parallel on the same samples) was tested by the Kappa (κ) index test at a 95% confidence interval using Win Episcope 2.0. A variable called complementary sensitivity (CSe), which measures the additional detection efficacy of method 1 over method 2 when both have similar specificity, was calculated as 100 × (no. samples positive by method 1 and negative by method 2/total no. samples positive by method 2) [17].

Differences in piroplasm infection or not (binomial) by each species regarding age [cat. A (<1 yr), cat. B (1–2 yr), cat. C (>2 yr)] (categorical; three levels), flock [F1–F4] (categorical; four levels) and sampling season (categorical; three levels), were assessed by analysis of variance and least square means comparison with the Tukey-Kramer adjustment for multiple comparisons in the GLM procedure of the SAS statistical package version 9.1 (SAS Institute Inc., Cary, NC, USA). P values less than 0.05 were considered statistically significant.

### Ethical considerations

Samples were collected by clinical veterinarians as part of the usual screening scheme on farms and Spanish ethical guidelines and animal welfare regulations (RD 1201/2005) were strictly respected. All herd owners had given an informed consent prior to the study.

## Results

### Optimization of the Luminex assay

#### Probe design

Firstly, the panel of probes designed for ovine piroplasms identification by RLB hybridisation (*B. ovis*, *B. motasi*, *T. ovis*, *T. luwenshuni*/OT1, *Theileria* sp. OT3 and *T. lestoquardi*) [2] was tested in the Luminex system. Three of them (*T. ovis*, *T. luwenshuni*/OT1 and *Theileria* sp. OT3) showed good specificity and sensitivity, but those for *B. ovis*, *B. motasi* and *T. lestoquardi* produced very low MFI values (low sensitivity) and cross-reactions (lack of specificity). Therefore, new probes were specifically designed for Luminex for *B. ovis* and *B. motasi*. For *T. lestoquardi*, which shares 99.5% identity with *T. annulata* in the 18S rRNA gene sequence, the probe developed for the Luminex detection of *T. annulata* was used [13], and designated *T. annulata*/*T. lestoquardi* probe hereafter. The *Theileria*-*Babesia* conserved catch-all TB probe used in RLB hybridisation assays had already been tested in a Luminex suspension array [13].

#### Analytical sensitivity and specificity and diagnostic sensitivity

**Figure 1:**
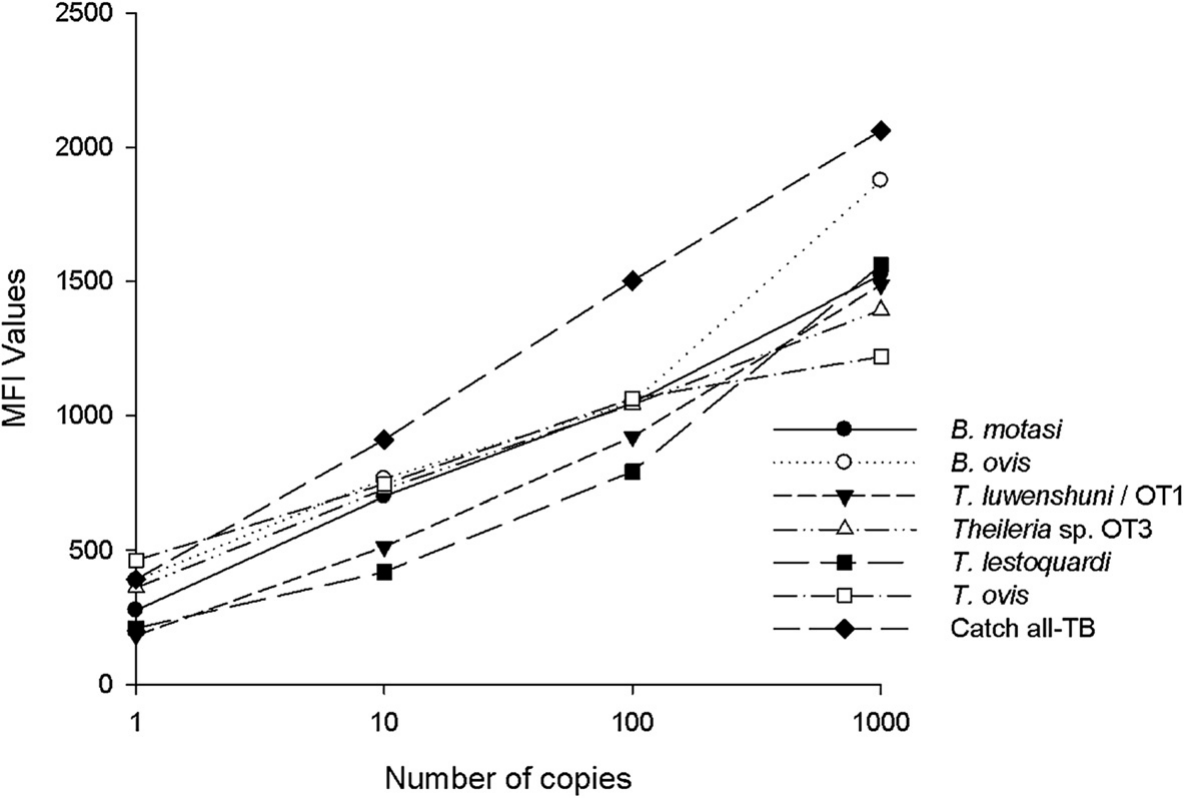
**Luminex detection of serially diluted plasmids (10**^**3**^**-1 copies) with the different *Theileria *and *Babesia *probes.** MFI, median fluorescent intensity; catch-all TB, *Theileria*-*Babesia* conserved probe.

Serial dilutions of single and mixed recombinant plasmids were amplified, digested with Lambda exonuclease, and run in triplicate in a Luminex experiment containing probes for *B. ovis*, *B. motasi*, *T. ovis*, *T. luwenshuni*/OT1, *Theileria* sp. OT3, *T. lestoquardi*/*T. annulata* and the catch-all TB probe. All were correctly identified by their specific probes and no cross-reactions were observed. The MFI values obtained in the Luminex multiplex assay for each probe and their corresponding plasmid dilutions are shown in Figure 1.

The detection limit of the Luminex assay was determined using serial dilutions of infected blood from an animal with a known level of *B. ovis* parasitaemia. The lowest parasitaemia that could be detected by the Luminex method corresponded to 0.35 parasites per μl of blood. Sequencing analysis of the amplification product obtained confirmed the presence of *B. ovis* DNA in this last dilution positive by Luminex.

#### Performance compared with RLB hybridisation

The performance characteristics of the Luminex assay were then assessed by testing 177 ovine blood samples. Aliquots of PCR amplified samples were analyzed in parallel by RLB and Luminex. Results showed very good concordance between techniques (κ = 0.938) for piroplasms detection with the catch-all TB probe. Thus, concordant results were obtained for 172 of the 177 specimens tested, 112 of those being positive by both techniques and 60 negative; the remaining five corresponded to samples positive by Luminex but undetected by RLB (Table 2). When considering each specific probe separately, best concordance was found for *B. ovis* probe (κ = 1.000), although only one sample was positive by both techniques. Also very good concordance was obtained for *T. ovis* and *Theileria* sp. OT3 which produced kappa values of 0.962 and 0.886, respectively, and good for *T. luwenshuni*/OT1 (κ = 0.677) and *B. motasi* (κ = 0.684). In all cases, disagreement was due to the higher number of samples detected by Luminex, so that overall, the diagnostic sensitivity was significantly higher, as indicated by the CSe of Luminex over RLB (Table 2).

**Table 2:** Luminex assay performance for the detection and identification of ovine piroplasms in sheep blood field samples compared with RLB hybridisation

**Probe**			**RLB**			**Kappa (κ)**	**CSe (%) Luminex**
		**Luminex**	**NEG**	**POS**	**Total**		
Catch-all TB		NEG	60	0	60		
		POS	5	112	117		
	Total		65	112	177	0.938 ± 0.027	4.46
*B. ovis*		NEG	176	0	176		
		POS	0	1	1		
	Total		176	1	177	1.000 ± 0.000	0.00
*B. motasi*		NEG	164	0	164		
		POS	6	7	13		
	Total		170	7	177	0.684 ± 0.120	85.71
*T. ovis*		NEG	117	0	117		
		POS	3	57	60		
	Total		120	57	177	0.962 ± 0.022	5.26
*T*. *luwenshuni*/OT1		NEG	162	0	162		
		POS	7	8	15		
	Total		169	8	177	0.677 ± 0.113	87.50
*Theileria* sp. OT3		NEG	93	0	93		
		POS	10	74	84	0.886 ± 0.035	13.51
	Total		103	74	177		

### Piroplasms distribution among clinically healthy sheep grazing in communal mountain pastures

Throughout the 2-year sampling period a total of 446 blood samples were collected, and 214 (48.0%) hybridised with the catch-all TB probe and with at least one of the ovine species-specific probes included in the Luminex assay (Table 3). Five different piroplasms were identified: *T. ovis*, *T. luwenshuni*/OT1, *Theileria* sp. OT3, *B. ovis* and *B. motasi*. Nearly half of the positive samples (49.1%, 105/214) corresponded to mixed infections, 52.4% of them (55/105) including both genera, *Babesia* and *Theileria*. However, the combination most commonly detected comprised *T. ovis* and *Theileria* sp. OT3 (31 samples). Only once were both babesia species, *B. ovis* and *B. motasi*, detected together as a mixed infection. *Theileria* sp. OT3 was the most frequently found piroplasm during the two sampling seasons (34.8%), second in detection was *T. ovis* (20.9%) whereas *B. motasi*, *T. luwenshuni*/OT1 and *B. ovis* were detected at lower prevalence (12.3%, 10.5% and 6.3%, respectively).

**Table 3:** Percentage of samples positive to piroplasms (single or mixed infection) and percentage of samples where each piroplasm species was identified

**FLOCK**	**Total analysed**	**% piroplasm positive**	**Species identified (%)**				
			***B. ovis***	***B. motasi***	***T. ovis***	***T. luwenshuni/*OT1**	***Theileria *sp. OT3**
F1	152	43.4	7.9	3.9	0.7	9.2	41.4
F2	149	20.8	2.7	10.1	1.3	0.7	10.7
F3	75	93.3	0.0	5.3	77.3	8.0	64.0
F4	70	67.1	17.1	42.9	45.7	37.1	40.0
Total	446	48.0	6.3	12.3	20.9	10.5	34.8

**Figure 2:**
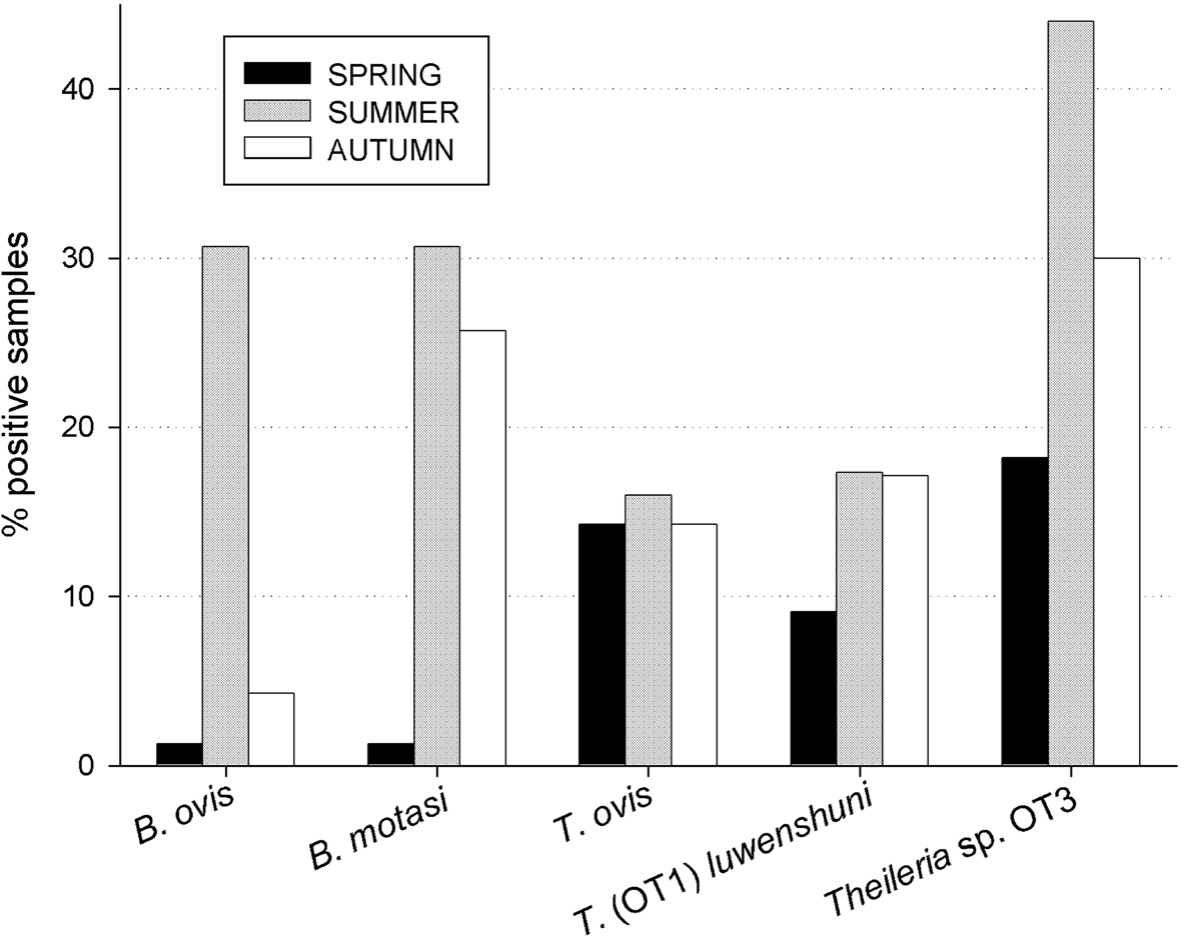
Seasonal distribution of the different piroplasms.

Infection with *T. ovis* (R^2^ = 0.700) and *T. luwenshuni*/OT1 (R^2^ = 0.492) were associated to flock, age and their combined effect, whereas infection with *Theileria* sp. OT3 (R^2^ = 0.586), *B. ovis* (R^2^ = 0.423) and *B. motasi* (R^2^ = 0.501) were associated to flock, age, and also sampling season and their combined effects. Thus, differences were found among flocks, with the lowest proportion of positive samples in F2 (p < 0.001). The highest was recorded in F3 particularly due to the high prevalence of *T. ovis* (p < 0.001). In all flocks except F2 prevalence of *Theileria* spp.-positive animals was significantly higher than that of *Babesia* spp. infections (p < 0.05). Except *B. ovis*, all other ovine piroplasm species were present in all sampled flocks (Table 3); *B. ovis* was detected in three of them, at a significantly lower proportion in F2 (p < 0.05). The highest proportion of *B. motasi*-positive samples was found in F4 (p < 0.0001). All ovine piroplasms were detected at the 3 sampling times (spring, summer and autumn), but differences among seasons were observed (Figure 2), being summer the season with the highest proportion of positive samples and spring the lowest (p = 0.0020). This was particularly clear for *Theileria* sp. OT3, *B. ovis* and *B. motasi*.

**Figure 3:**
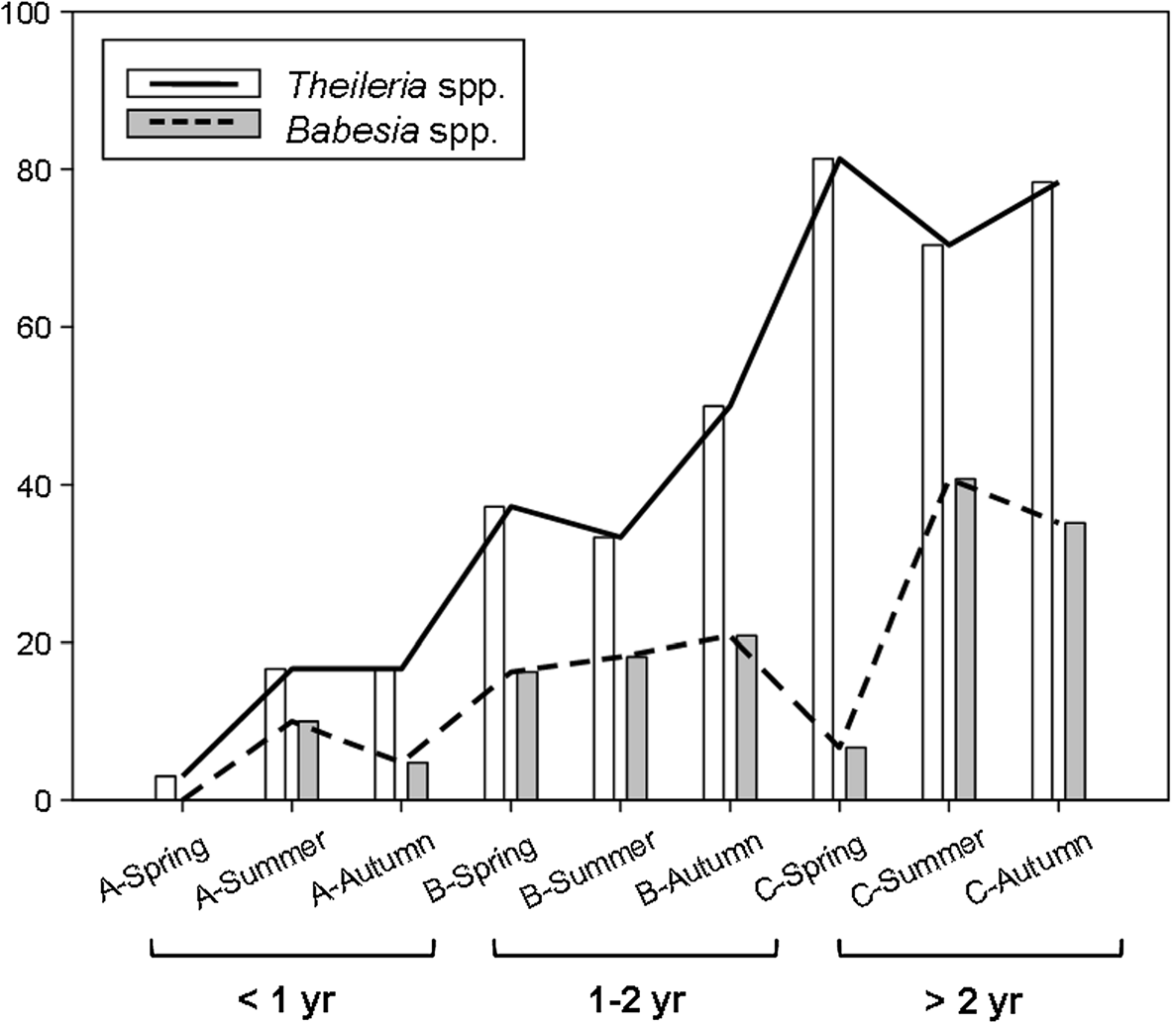
**Distribution of *Theileria *spp. and *Babesia *spp. infection according to age and sampling season.** Percentage of positive samples at each sampling season grouped by age is represented by bars; the lines would represent the expected evolution of infection in one animal as time goes by.

Proportion of piroplasm-positive animals increased with age. This was particularly clear for animals infected with *Theileria* species, where significant differences were observed among the three age categories (p < 0.0001). Distribution of infection with *Babesia* spp. varied significantly between category A and the other two age categories (p < 0.0001), but only marginally between categories B and C (p = 0.0711). When considering age and season together, an increasing trend was observed in the proportion of animals infected with piroplasms so that the longer the exposure to ticks the higher the prevalence (Figure 3). All except one lamb (Cat. A) in F4 were negative in spring. However, whereas in F1 and F2 lambs remained mainly negative for the whole grazing period, infection was already detected in summer in F3 and F4 (data not shown). In spring, hoggets (Cat. B) were still negative in F1 and F2 but infection was widespread in F3 and F4; as the sampling period progressed and exposure to the tick vectors increased, proportion of positive animals increased in all flocks, both in hoggets and adult ewes (Cat. C). This increment was clearer for *Theileria* spp.-infected animals, whereas *Babesia* spp. infections showed a decrease at the end of the grazing season that increased again after the following grazing season.

### Ticks collected from the animals and the vegetation

During the second sampling season, a total of 192 ticks (125 adults, 60 nymphs and 7 larvae) were removed from 59 of the 222 animals examined. The mean number of ticks removed per infested animal was 3.5. However, most of the ticks (70%) were collected from a single flock (F4), where 30% of the animals were infested with an average of 6.4 ticks per infested animal. Four different tick species were found and identified as *Ixodes ricinus* (43.8%), *Haemaphysalis punctata* (34.9%), *Rhipicephalus bursa* (15.1%) and *Dermacentor marginatus* (6.3%). All *D. marginatus* (100%) and almost all *H. punctata* (95.5%) collected from the animals were adults, whereas the three tick stages of *R. bursa* and *I. ricinus* were collected. Still, *R. bursa* adults were most commonly collected (65.5%), all of them being collected in summer. Conversely, nymphs accounted for 59.5% of all *I. ricinus*, and were mainly collected in autumn. *H. punctata* was also most commonly collected in autumn than in any other season.

**Figure 4:**
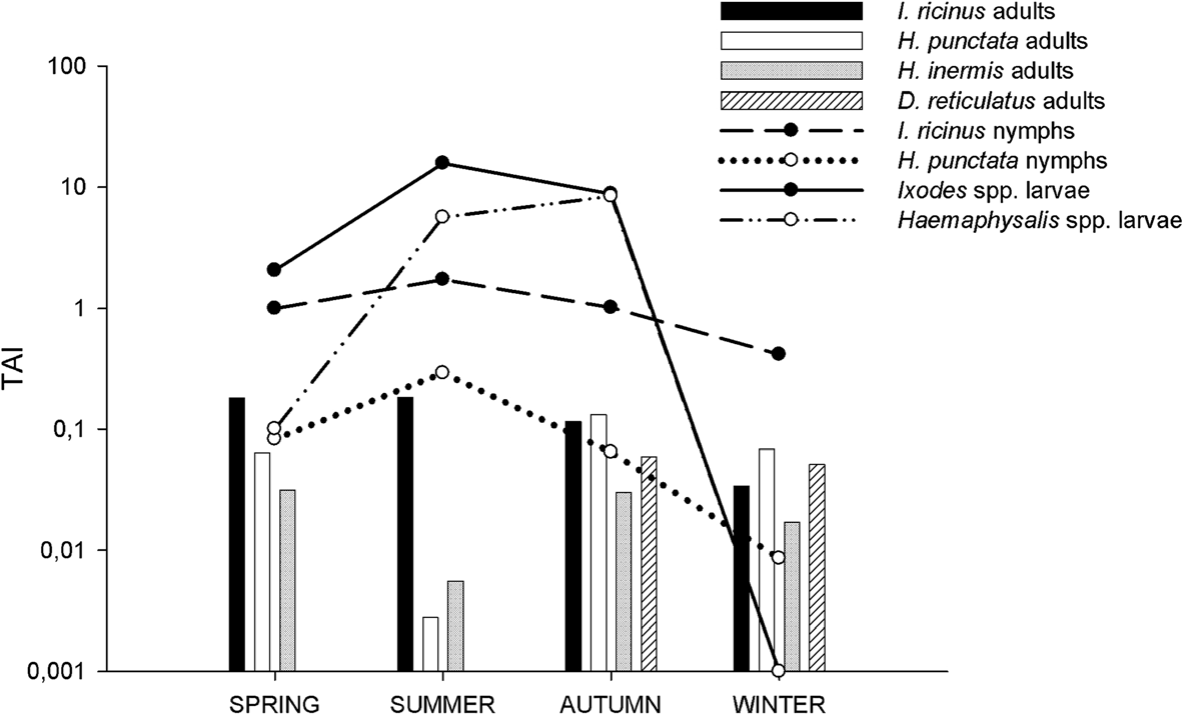
**Seasonal abundance of the different stages of the most abundant tick species.** Tick abundance indexes (TAI) represent the number of each tick species and stage collected in transects of 100 m^2^, and are calculated as TAI = TR x 100 / *a*, where TR is the number of ticks recorded and *a* is the sampled area in square meters.

A total of 10,943 ticks were collected (275 adults, 1,148 nymphs and 9,520 larvae) from the vegetation over the 2-year study. Captured adult ticks belonged to 8 different species, being more abundant *I. ricinus* (159), *H. punctata* (69), *Dermacentor reticulatus* (19) and *Haemaphysalis inermis* (22). Sporadic captures of *D. marginatus* (2), *Ixodes frontalis* (2) and *R. bursa* (1) and *Haemaphysalis sulcata* (1) were made but were not included for further analysis. For immature stages, except for one *I. frontalis*, all identified nymphs belonged to the species *I. ricinus* (1,003) and *H. punctata* (144), and similarly, all larvae belonged to the genera *Ixodes* and *Haemaphysalis* (7,012 and 2,508, respectively). There were differences in the seasonal patterns according to the tick species and stages (Figure 4). Thus, adults of *I. ricinus* showed a similar activity throughout the year, with a slight decrease in winter, adults of *H. punctata* were less abundant in summer, and *H. inermis* and *D. reticulatus* adults were only captured in cooler seasons (autumn and winter). Regarding immature stages, seasonal pattern of *I. ricinus* and *H. punctata* larvae and nymphs was nearly overlapping, with the highest abundance in summer and autumn and decreasing thereafter, until the activity of larvae of both species practically disappeared in winter.

## Discussion

Little attention has been given to ovine piroplasmosis compared to bovine piroplasmosis despite its widespread distribution in southern Europe, the Middle East, China, and through tropical and subtropical areas. New techniques with higher sensitivity and large multiplexing capacity for the detection of carrier animals are necessary to better understand the epidemiology of ovine piroplasmosis. The Luminex® xMAP procedure presented here is a high throughput technique that combines large multiplexing capacity with high sensitivity, making it an ideal tool for the detection and identification of single and mixed piroplasm infections in sheep blood. As already shown for cattle [13], this study demonstrated the benefits of Luminex over RLB, another multiplex technique widely used for piroplasms detection and identification. Small modifications in probe design for *B. ovis* and *B. motasi* were needed to transfer the piroplasm RLB assay to the new hybridisation Luminex® xMAP platform. Thus, the new multiplex panel of ovine piroplasm probes included in the Luminex® xMAP assay allowed specific and sensitive detection of serial dilutions of a panel of recombinant plasmids (1 copy of target gene) for the different *Theileria* and *Babesia* species. Moreover, the Luminex® xMAP procedure was able to detected piroplasms in samples that were negative by RLB hybridisation. Finally, the inclusion of two control probes, the catch-all TB probe, that detects a wide spectrum of piroplasm species [2,18], and the IAC probe, that monitors for the presence of PCR inhibitors [13,19], assures that all new species and genotypes are detected at least at the group level and guarantees that false negatives do not occur.

Once validated, we used the Luminex® xMAP technology to monitor piroplasm infection throughout two years among clinically healthy sheep from four flocks grazing in the same communal mountain pasture in a region where subclinical piroplasm infection had been detected in the past [2]. In this study, the same piroplasm species were detected in the Basque Country, but prevalences were different to those observed previously. While the percentage of animals positive for *B. motasi* increased considerably in this study, *T. luwenshuni*/OT1 showed a marked decrease compared to the study carried out in 2004. Then, samples were selected to represent the ovine population of the whole Basque Country (Study I – [2]); here, only four flocks restricted to a very specific location were sampled. Despite sharing the same mountain communal pastures, differences were found among flocks, probably due to differences in management. In fact, in flocks F1 and F2, milking animals and replacement lambs, which represented *ca.* 85% of the flock census, remained until July in farmland pastures with scarce presence of ticks, before going to communal mountain pastures; in flocks F3 and F4, 50% of the animals in the flock grazed in mountain pastures from April. These differences in tick exposure periods, along with a more intensive management system in F1 and F2 as compared to F3 and F4 where management is more traditional, would explain differences in piroplasms infection.

Taking into account that lambing in Latxa sheep in Alava occurs in January-February, three age categories (A, <1 year; B, 1–2 years; C, >2 years) were established in such manner that during the first annual sampling (spring), lambs (Cat. A) from the four flocks had not yet been exposed to ticks. Therefore, they were expected to be negative in spring and acquire the infection while the sampling period progressed and exposure to the tick vectors increased. Animals in Cat. B (hoggets) and C (ewes) had been exposed to ticks one or more grazing seasons, respectively, when sampled in spring. The analysis of piroplasm infection considering age at sampling and sampling season, showed increasing piroplasm prevalence with increasing age of animals and tick exposure. The increasing trend observed could be attributed to continued re-infection associated with re-exposure to ticks at grazing. Still, in the case of carrier animals infected with *Babesia* spp., where an intra-leukocytic phase does not occur, parasitaemia is much lower and sometimes barely perceptible [8]. Hence, *Babesia* spp.-infected animals were more easily detected after flocks were re-exposed to infected ticks at grazing.

In order to evaluate the evolution of piroplasm infection in relation with tick abundance and distribution, ticks were removed from sampled animals and collected from the vegetation in the areas where sheep graze. The number of ticks collected from the animals was too low to draw many conclusions. Still, the natural vectors of ovine piroplasms, *H. punctata* and *R. bursa*, were collected from the animals at some point over the sampling year. The most abundantly collected tick species, both from the animals and the vegetation, was *I. ricinus* but abundance of questing *I. ricinus* in this area was lower than that reported in other parts of the Basque Country [20,21]. This tick species has not been described as vector of any ovine piroplasm species, but DNA of *T. ovis*, *Theileria* sp. OT3 and *B. ovis* has been detected in *I. ricinus* questing ticks [22]. On the other hand, *H. punctata*, which is vector of *B. motasi* and *T. ovis*, was the second most abundantly collected tick from the animals and showed higher abundance in vegetation than previously reported [21]. Mainly adults were recovered from the animals and were mostly collected in autumn, the season when questing *H. punctata* adult ticks were also most active. Immature stages captured from the vegetation were more abundant in summer and autumn and decreased thereafter, until the activity of larvae disappeared in winter. In accordance with tick seasonality, summer and autumn would be the seasons of highest risk of infection with *B. motasi*. Accordingly, results showed that *B. motasi* infection was most prevalent in summer and autumn (data not shown), which could be a reason for concern considering the previously reported association between *B. motasi* and sheep abortion [2].

*R. bursa* transmits *T. ovis* and *B. ovis*[3,7]. In this study, *R. bursa* accounted for 15.1% of the ticks collected directly from the animals (adults in summer and, nymphs and larvae in autumn), but only 1 adult was collected by blanket dragging. Other studies carried out in the Basque Country, detected larger proportions of questing *R. bursa* (2-10% of adult ticks collected) but, same as here, adult *R. bursa* ticks were all collected at the end of spring and early summer with peaks in June and July [21,23]. Although *B. ovis* is described as the most virulent piroplasm species for sheep, here, animals positive to *B. ovis* did not show any clinical signs of disease at sampling. Incidence of *B. ovis* infection was particularly high in F4, as was infestation with *R. bursa*. In this flock, lambs are exposed to ticks for the first time at early spring, before maternal antibodies wane [24]. Therefore, lambs bitten by *B. ovis*-infected *R. bursa* adult ticks at the end of spring and early summer would not develop disease but acquire protective immunity. Later, in autumn, when adults of *R. bursa* are not active, exposure to infected larvae and nymphs, would favour premunition [10]. Due to differences in management, lambs in F1 and F2 start grazing in mountain pastures in July, therefore they have less probability of being bitten by *R. bursa* adult ticks. This could explain why they remained negative at sampling not only in summer but also in autumn. In the case of *T. ovis*, which as other *Theileria* spp., is detectable in blood for long periods, associations between the peak activity of vectors and host infection are more difficult to establish. Besides, *T. ovis* can be transmitted by both *R. bursa* and *H. punctata*. In any case, *T. ovis* is considered non-pathogenic and distinct clinical signs are rare [3]. The principal vector of *T. luwenshuni* in China has been reported to be *Haemaphysalis qinghaiensis*[6], suggesting that another *Haemaphysalis* species could be involved in the transmission of *T. luwenshuni*/OT1 in Spain. In a previous study, *Theileria* sp. OT1 was detected in 3 different tick species, *I. ricinus*, *H. punctata* and *D. reticulatus*[22]. The vector of *Theileria* sp. OT3 remains unknown, but a possible association with *H. punctata* was proposed [22].

## Conclusions

In conclusion, this study confirmed a situation of endemic stability for piroplasm infection in the region, where infection is present in the absence of clinical signs, and mountain grazing allows for sufficient inoculation rates to maintain such a situation [25]. Although mild or sporadic cases of clinical disease might go unnoticed during mountain grazing, when animals are not closely monitored, absence of clinical signs at sampling could be due to the fact that in endemic areas livestock can develop resistance to ticks and piroplasm infection [26,27]. Future studies on the presence of piroplasms in the collected questing ticks will provide more information on the proportion of infection of the different combinations between piroplasm species and vector tick. For these studies, the Luminex® xMAP technology using probes for ovine piroplasms as described here, probes for piroplasm species that infect cattle as previously reported [13] and probes for horses currently under development, will facilitate rapid and abundant data in a multiplexing and highly sensitive format of high processability.

## Competing interests

The authors declare that they have no competing interests.

## Authors’ contributions

AR carried out the experimental work and participated in drafting of the manuscript. JFB carried out tick collection and identification. ALG collaborated in the design of the study, interpreted data and critically revised the manuscript. RAJ performed the statistical analysis and participated in the critical reading of the publication. AH conceived the study, participated in its design and coordination, and drafted the final manuscript. All authors read and approved the final manuscript.

## References

[B1] Gabiña D Arrese F Arranz J Beltran de Heredia I Average milk yields and environmental effects of Latxa sheep J Dairy Sci 1993 76 1191 1198 10.3168/jds.S0022-0302(93)77448-2

[B2] Nagore D García-Sanmartín J García-Pérez AL Juste RA Hurtado A Identification, genetic diversity and prevalence of *Theileria* and *Babesia* species in a sheep population from Northern Spain Int J Parasitol 2004 34 1059 1067 10.1016/j.ijpara.2004.05.008 15313132

[B3] Preston PM Service M.W Theilerioses Encyclopedia of arthropod-transmitted infections of man and domesticated animals 2001 Wallingford, UK: CABI Publishing 487 502

[B4] Uilenberg G *Babesia*-A historical overview Vet Parasitol 2006 138 3 10 10.1016/j.vetpar.2006.01.035 16513280

[B5] Yin H Luo J Schnittger L Lu B Beyer D Ma M Guan G Bai Q Lu C Ahmed J Phylogenetic analysis of *Theileria* species transmitted by *Haemaphysalis qinghaiensis* Parasitol Res 2004 92 36 42 10.1007/s00436-003-0900-z 14598167

[B6] Yin H Luo J Guan G Lu B Ma M Zhang Q Lu W Lu C Ahmed J Experiments on transmission of an unidentified *Theileria* sp. to small ruminants with *Haemaphysalis qinghaiensis* and *Hyalomma anatolicum anatolicum* Vet Parasitol 2002 108 21 30 10.1016/S0304-4017(02)00166-8 12191896

[B7] Uilenberg G Service M.W Babesiosis Encyclopedia of arthropod-transmitted infections of man and domesticated animals 2001 Wallingford, UK: CABI Publishing 53 60

[B8] Sonenshine DE Biology of ticks, Volume II 1993 New York: Oxford University Press

[B9] Chauvin A Moreau E Bonnet S Plantard O Malandrin L Babesia and its hosts: adaptation to long-lasting interactions as a way to achieve efficient transmission Vet Res 2009 40 37 10.1051/vetres/2009020 19379662 PMC2695028

[B10] Yeruham I Hadani A Galker F Some epizootiological and clinical aspects of ovine babesiosis caused by Babesia ovis–a review Vet Parasitol 1998 74 153 163 10.1016/S0304-4017(97)00143-X 9561703

[B11] Liu AH Yin H Guan GQ Schnittger L Liu ZJ Ma ML Dang ZS Liu JL Ren QY Bai Q Ahmed JS Luo JX At least two genetically distinct large *Babesia* species infective to sheep and goats in China Vet Parasitol 2007 147 246 251 10.1016/j.vetpar.2007.03.032 17531391

[B12] Schnittger L Yin H Qi B Gubbels JM Beyer D Niemann S Jongejan E Ahmed JS Simultaneous detection and differentiation of *Theileria* and *Babesia* parasites infecting small ruminants by reverse line blotting Parasitol Res 2004 92 189 196 10.1007/s00436-003-0980-9 14652747

[B13] Ros-García A Juste RA Hurtado A A highly sensitive DNA bead-based suspension array for the detection and species identification of bovine piroplasms Int J Parasitol 2012 42 207 214 10.1016/j.ijpara.2011.12.001 22233830

[B14] Gil-Collado J Guillén-Llera JL Zapatero-Ramos LM Claves para la identificación de los Ixodoidea españoles (adultos) Rev Iber Parasitol 1979 39 107 111

[B15] Manilla G Fauna D’Italia: Acari, Ixodida 1998 Bologna: Edizioni Calderini

[B16] Georges K Loria GR Riili S Greco A Caracappa S Jongejan F Sparagano O Detection of haemoparasites in cattle by reverse line blot hybridisation with a note on the distribution of ticks in Sicily Vet Parasitol 2001 99 273 286 10.1016/S0304-4017(01)00488-5 11511414

[B17] Juste RA Garrido JM Geijo M Elguezabal N Aduriz G Atxaerandio R Sevilla I Comparison of blood polymerase chain reaction and enzyme-linked immunosorbent assay for detection of *Mycobacterium avium* subsp. *paratuberculosis* infection in cattle and sheep J Vet Diagn Invest 2005 17 354 359 10.1177/104063870501700409 16130994

[B18] Gubbels JM de Vos AP van der Weide M Viseras J Schouls LM de Vries E Jongejan F Simultaneous detection of bovine *Theileria* and *Babesia* species by reverse line blot hybridization J Clin Microbiol 1999 37 1782 1789 10325324 10.1128/jcm.37.6.1782-1789.1999 PMC84950

[B19] Hoorfar J Malorny B Abdulmawjood A Cook N Wagner M Fach P Practical considerations in design of internal amplification controls for diagnostic PCR assays J Clin Microbiol 2004 42 1863 1868 10.1128/JCM.42.5.1863-1868.2004 15131141 PMC404670

[B20] Barandika JF Hurtado A Juste RA García-Pérez AL Seasonal dynamics of *Ixodes ricinus* in a 3-year period in northern Spain: first survey on the presence of tick-borne encephalitis virus Vector Borne Zoonotic Dis 2010 10 1027 1035 10.1089/vbz.2009.0148 20455780

[B21] Barandika JF Olmeda SA Casado-Nistal MA Hurtado A Juste RA Valcarcel F Anda P García-Pérez AL Differences in questing tick species distribution between Atlantic and continental climate regions in Spain J Med Entomol 2011 48 13 19 10.1603/ME10079 21337943

[B22] García-Sanmartín J Barandika JF García-Pérez AL Juste RA Hurtado A Distribution and molecular detection of *Theileria* and *Babesia* in questing ticks from Northern Spain Med Vet Entomol 2008 22 318 325 10.1111/j.1365-2915.2008.00748.x 19120958

[B23] Barandika JF Berriatua E Barral M Juste RA Anda P García-Pérez AL Risk factors associated with ixodid tick species distributions in the Basque region in Spain Med Vet Entomol 2006 20 177 188 10.1111/j.1365-2915.2006.00619.x 16796614

[B24] Yeruham I Hadani A Galker F Rosen S A study of an enzootic focus of sheep babesiosis (Babesia ovis, Babes, 1892) Vet Parasitol 1995 60 349 354 10.1016/0304-4017(95)00783-7 8747918

[B25] Norval RAI Perry BD Young AS The Epidemiology of Theileriosis in Africa 1992 London, UK: Academic Press

[B26] Dalgliesh RJ Warren KS Babesiosis Immunology and molecular biology of parasitic infections 1993 3 Boston: Blackwell Scientific Publications

[B27] Wikel SK Tick modulation of host immunity: an important factor in pathogen transmission Int J Parasitol 1999 29 851 859 10.1016/S0020-7519(99)00042-9 10480722

